# Circadian Influences on the Habenula and Their Potential Contribution to Neuropsychiatric Disorders

**DOI:** 10.3389/fnbeh.2021.815700

**Published:** 2022-01-28

**Authors:** Callum J. Young, David Lyons, Hugh D. Piggins

**Affiliations:** School of Physiology, Pharmacology, and Neuroscience, University of Bristol, Bristol, United Kingdom

**Keywords:** circadian, depression, clock gene, burst firing, epithalamus

## Abstract

The neural circadian system consists of the master circadian clock in the hypothalamic suprachiasmatic nuclei (SCN) communicating time of day cues to the rest of the body including other brain areas that also rhythmically express circadian clock genes. Over the past 16 years, evidence has emerged to indicate that the habenula of the epithalamus is a candidate extra-SCN circadian oscillator. When isolated from the SCN, the habenula sustains rhythms in clock gene expression and neuronal activity, with the lateral habenula expressing more robust rhythms than the adjacent medial habenula. The lateral habenula is responsive to putative SCN output factors as well as light information conveyed to the perihabenula area. Neuronal activity in the lateral habenula is altered in depression and intriguingly disruptions in circadian rhythms can elevate risk of developing mental health disorders including depression. In this review, we will principally focus on how circadian and light signals affect the lateral habenula and evaluate the possibility that alteration in these influences contribute to mental health disorders.

## Introduction

It takes a mere cursory glance at social media to deduce that the mental-health conversation is as critical today as it has ever been. We live in a world where the public feel that mental illness remains an unsolved dilemma. They express dissatisfaction with their acute and long-term suffering, as well as the pharmacotherapies available to alleviate this (Priest et al., 1996; Hergerl et al., 2003; Partridge et al., 2012; Horowitz and Graf, 2019). Exemplifying this discontent, depression is a mood disorder characterised by persistent low mood and anhedonia (Tolles-Correia et al., 2018). This mental illness is the leading cause of disability globally (Charlson et al., 2019) and affects over 264 million people (GBD 2017 Disease Injury Incidence Prevalence Collaborators, 2018). Despite this, a universally effective treatment for depression is unavailable, and approximately one third of patients fail to respond to conventional antidepressant drugs (Corriger and Pickering, 2019). Moreover, there is an absence of consensus on how to define depression (Tolles-Correia et al., 2018), making its diagnosis and treatment one of the greatest challenges in modern psychiatry.

As such, a surge of neuroscience research continues to dissect the pathophysiology that underpins depression. At the turn of the previous decade, the brain's epithalamus was identified as a potential new therapeutic target for treatment resistant depression. Specifically, deep brain stimulation (DBS) of the lateral habenula (LHb) was shown to alleviate depressive symptoms in a therapy-refractive patient (Sartorius et al., 2010). Further, when DBS was discontinued, there was an immediate and profound relapse of these depressive symptoms. This highlights the potential importance of the epithalamus in mood regulation (Shabel et al., 2014).

The LHb is an evolutionarily conserved epithalamic structure (Bianco and Wilson, 2009; Hikosaka, 2010), most investigated for its role as an “anti-reward” centre (Shabel et al., 2012) with anatomical connections allowing it to exert inhibitory control over midbrain monoaminergic centres (Hu et al., 2020). In part, it is these network connections which make the LHb an interesting candidate for depression research (Sartorius and Henn, 2007). Recently, rodent studies have further implicated the epithalamus in depression (Hu et al., 2020). More specifically, elevated neural activity in the rodent LHb has been associated with various depression models including learned helplessness (Li et al., 2011; Cui et al., 2018), chronic stress (Cerniauskas et al., 2019) and chronic pain (Zhuo et al., 2019). A landmark paper has also implicated the LHb in the antidepressant mechanism of ketamine (Yang et al., 2018a), although this utilised supra-clinical doses. In addition, human fMRI studies show differing LHb activity, and functional connectivity, between depressed and healthy individuals (Lawson et al., 2017; Zhu et al., 2019; Rivas-Garajales et al., 2021). From this evidence, it is implied that the LHb is an epicentre for depression's mechanistic underpinnings.

In addition to this, the LHb is situated within an extended neural circadian circuit (Bano-Otalora and Piggins, 2017). Immunohistochemical evidence indicates that the rodent LHb receives regulation from the master circadian pacemaker in the suprachiasmatic nucleus (SCN; Hastings et al., 2018). Further, the LHb receives input from other hypothalamic circadian oscillators (Guilding and Piggins, 2007; Guilding et al., 2009; Poller et al., 2013; Stamatkis et al., 2016) and the retina (Qu et al., 1996). Indeed, rodent LHb neurones show diurnal variation in their electrophysiological properties and are responsive to retinal illumination (Zhao and Rusak, 2005; Sakhi et al., 2014b).

Interestingly, circadian disruptions are commonly reported in patients suffering from depression (Jagannath et al., 2013; Difrancesco et al., 2019). Moreover, the efficacy of certain antidepressant therapies has been hypothesised to be time of day dependent (Swanson et al., 2017). Given its endogenous circadian properties, and neural connections with brain structures associated with circadian timing and mood, the LHb is a likely candidate as a locus of interaction between depression and circadian disruptions. In this review, we evaluate the current state of understanding regarding the LHb's anatomical and molecular organisation, and its functional involvement in the pathology of depression. We also consider the intrinsic and extrinsic factors shaping the daily variation in this structure's properties, and explore the potential links between circadian rhythms, depression, and the LHb.

## Organisation of the Habenula Complex

The habenula is a cellularly diverse structure located in the posterior-dorsal-medial region of the thalamus (Hu et al., 2020) and can be divided into anatomically distinct lateral and medial nuclei [the LHb and the medial habenula (MHb); Diaz et al., 2011]. The LHb and MHb differ in their afferent and efferent connections (Aizawa et al., 2011, 2012) and play largely different roles in their respective neural circuits. Unlike the predominantly glutamatergic LHb, the MHb contains substance P expressing neurones and cholinergic cells in its dorsal and ventral division, respectively (Aizawa et al., 2012). Although both structures are becoming increasingly implicated in the pathophysiology of various psychiatric conditions (Metzger et al., 2021), the nature of these differ. The MHb is predominantly implicated in nicotine withdrawal (Fowler et al., 2011; Hsu et al., 2013), drug addiction (Glick et al., 2006; Lopez et al., 2018) and anxiety (Zhang et al., 2016), while functional studies suggest that aberrant LHb activity is more closely associated with depression (Li et al., 2013; Seo et al., 2018; Yang et al., 2018b). However, recent research has highlighted an increasingly complex role for the LHb in various reward pathologies, including drug seeking (Li et al., 2017; Nair et al., 2021).

**Figure 1:**
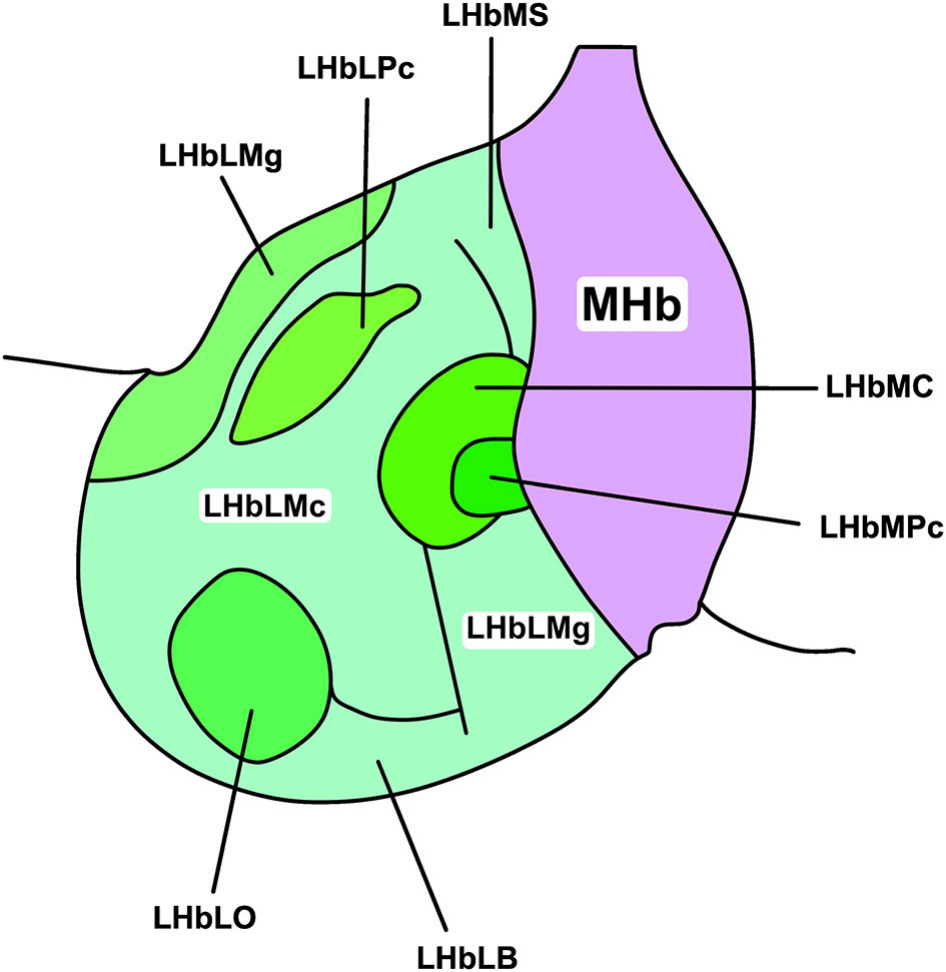
Subnuclear organisation of the rat LHb. The habenular complex can be divided into lateral and medial subdivisions (LHb and MHb, respectively). The LHb can be further divided into medial and lateral regions (LHbL and LHbM, respectively), and thus into nine cytochemically, topographically, and morphologically distinct subnuclei. These include, in the LHbL: the marginal subnucleus of the LHbL (LHbLMg), the magnocellular subnucleus of the LHbL (LHbLMc), the oval subnucleus of the LHbL (LHbLO), the basal subnucleus of the LHbL (LHbLB), and the parvocellular subnucleus of the LHbL (LHbLPc). In the LHbM: the superior subnucleus of the LHbM (LHbMS), the central subnucleus of the LHbM (LHbMC), the parvocellular subnucleus of the LHbM (LHbMPc), and the marginal subnucleus of the LHbM (LHbMMg). Redrawn and adapted from Wagner et al. (2014).

The LHb shows considerable intranuclear heterogeneity in morphology and cytochemistry (Diaz et al., 2011; Hu et al., 2020). As such, it can be subdivided into medial and lateral divisions (LHbM and LHbL; Aizawa et al., 2012) and further apportioned into nine subnuclei based on immunocytochemical, topographical, and morphological criteria (Figure 1; Wagner et al., 2014). Subsequent transcriptomic profiling suggests though that LHb cell types are heterogeneous and do not map closely with these subnuclear boundaries (Wagner et al., 2016). Instead, topographically and transcriptionally distinct cell clusters have been identified as a more useful approach to divide the LHb (Hashikawa et al., 2020). Not only does this approach provide a more accurate reflection of cell type-distribution, but these transcriptionally distinct neurones are differentially recruited by aversive stimuli (Cerniauskas et al., 2019), illustrating a heterogeneous response to stress within the LHb.

As well as defining LHb cell types according to their transcriptome, neurones can be distinguished by their mode of firing (Weiss and Veh, 2011; Sakhi et al., 2014b). Three modes of firing have been identified in the LHb: silent, tonic firing, and burst firing (Yang et al., 2018a). Another study distinguished between depolarised and hyperpolarised silent states and described an additional bistable state in which neurones oscillate between firing and quiescence (Sakhi et al., 2014b). Cells exhibiting different firing modes have different resting membrane potentials (RMP), with bursting neurones more hyperpolarised than their tonic, silent, and bistable counterparts. Firing mode is not dependent on cellular morphology or subnuclear location, but is acutely sensitive to perturbations in membrane potential (Kim and Chang, 2005; Weiss and Veh, 2011). Indeed, LHb neurones show extensive firing mode plasticity, with depolarising stimuli driving a shift in firing mode from burst to tonic firing (Cui et al., 2018), and injections of negative current causing rebound bursts (Kim and Chang, 2005; Yang et al., 2018a).

**Figure 2:**
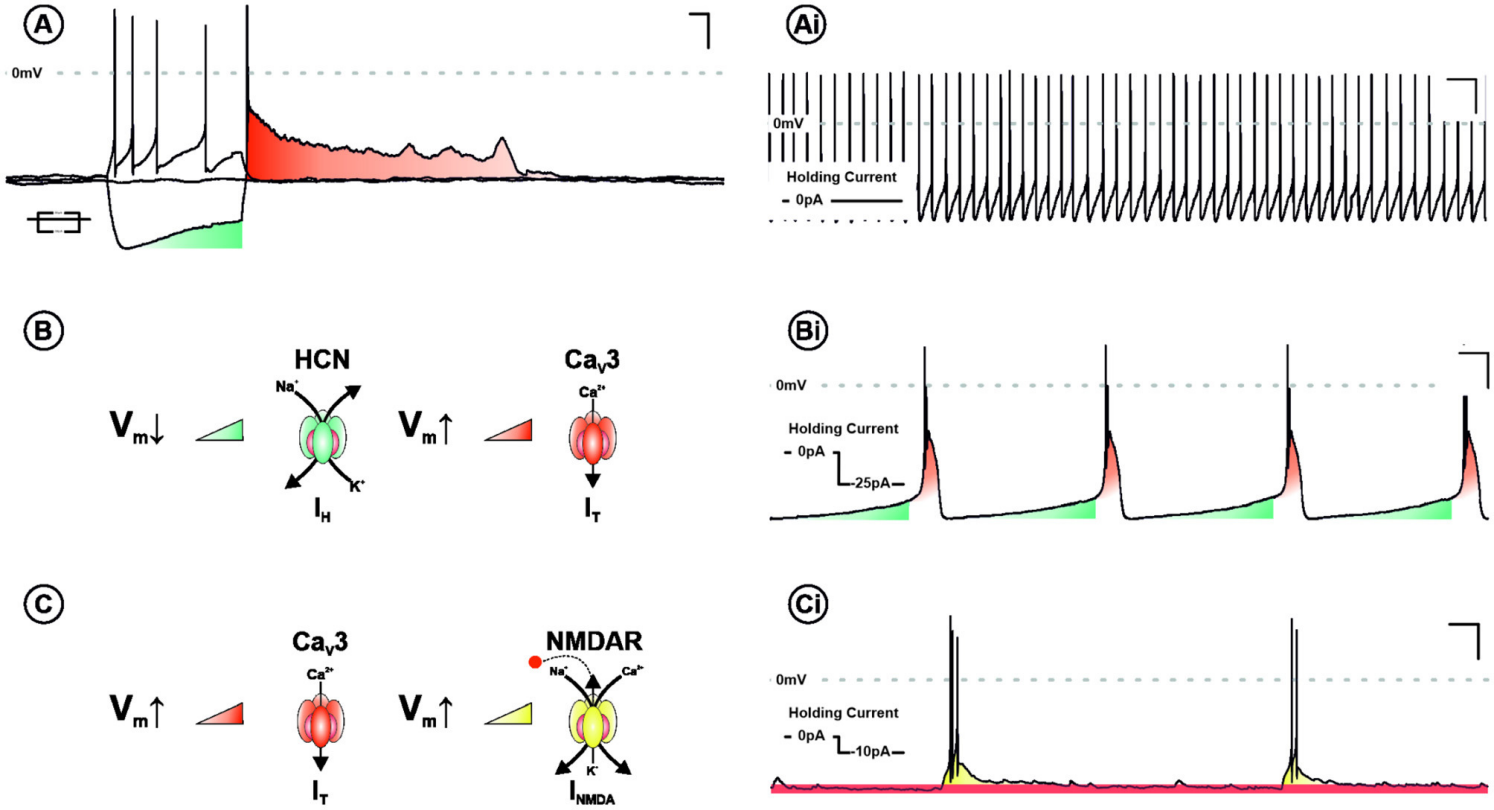
Ionic mechanisms underpinning burst firing in the LHb. **(A)** Voltage response of a LHb neurone to negative and positive square form current injection. Note the depolarising sag (blue) and subsequent rebound excitation (red). In addition to tonic discharge patterns **(Ai)**, LHb neurones are capable of two mechanistically distinct modes of burst firing. Both require membrane hyperpolarisation and are characterised by rhythmic periods of depolarisation, capped by high frequency action potentials. One mechanism, requiring more pronounced depolarisation, is cell autonomous and recurrent **(B)**. This cycle relies on the sequential activation and de-inactivation of hyperpolarisation-activated cyclic nucleotide–gated channels (HCN—blue) and T-type calcium channels (Ca_V_3—red), respectively **(Bi)**. The other is network dependent **(C)** and occurs within a specific membrane potential range (−55 to −65 mv). This depends on the interplay between Ca_V_3 and NMDA receptors (NMDARs—Yellow). Activation of T-type removes Mg^2+^-dependent blockade of NMDARs, synergistically driving a burst of action potentials. With membrane potential repolarisation Ca_V_3 channels de-inactivate permitting the cycle to begin again **(Ci)**.

The necessity for hyperpolarisation in burst firing neurones is explained by two principal bursting mechanisms in the LHb (Yang et al., 2018a). The first—requiring the most pronounced hyperpolarisation—is cell autonomous and driven by sequential recruitment of intrinsic ionic conductances. These are hyperpolarisation-activated cyclic nucleotide–gated channels and T-type calcium channels (TTCCs), which are activated and de-inactivated by membrane hyperpolarisation, respectively. The second occurs within a specific membrane potential range (−55 to −65 mV), and is network dependent, utilising the interplay between NMDA receptors and TTCCs. Understanding the electrophysiological mechanisms underpinning burst firing is critical to elucidating the function of this activity profile (Figure 2).

**Figure 3:**
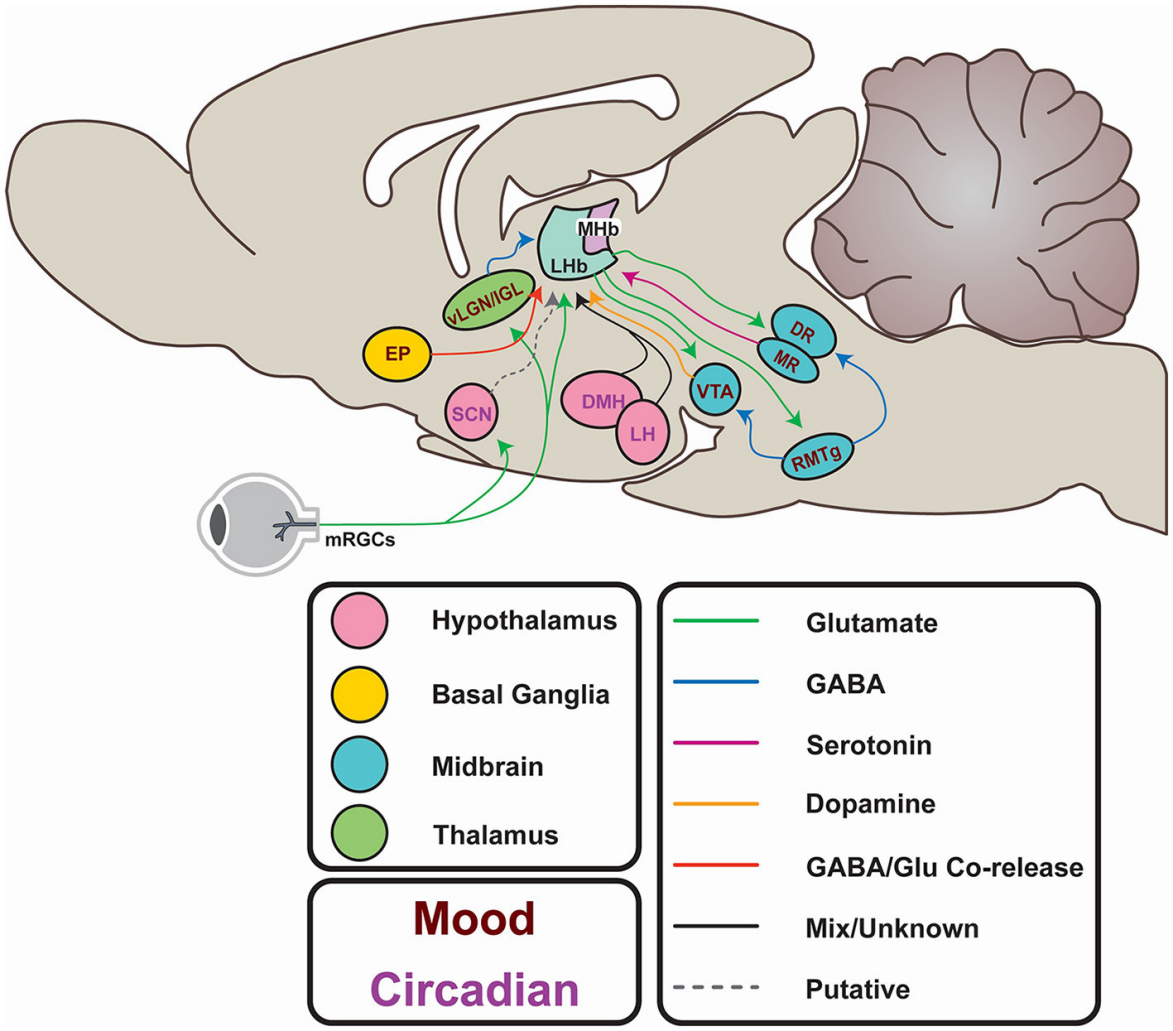
Afferents and efferents of the LHb. From the hypothalamus, the lateral hypothalamic area (LH) and dorsomedial hypothalamus (DMH) potentially entrain the LHb to circadian rhythms. These areas receive input from the suprachiasmatic nucleus (SCN), which in turn may entrain the LHb, either directly (*via* putative Prokineticin 2-containing efferents) or indirectly *via* SCN innervation of the LH and DMH. The SCN itself is entrained to the external light-dark cycle through photic information directly conveyed by non-image forming retinal projections. Similar projections from melanopsin-expressing retinal ganglion cells (mRGCs) terminate in areas immediately adjacent to the LHb. Additionally, the LHb may receive photic information *via* the ventral lateral geniculate or Intergeniculate nucleus (vlGN/IGL). Input from the entopeduncular nucleus (EP) encodes anti-reward, and activity in this pathway is elevated in depression. Excitatory projections to the rostromedial tegmental area (RMTg) provides feedforward inhibition to the ventral tegmental area (VTA) and the raphe nuclei (DR and MR) which regulates mood and reward learning. The VTA provides reciprocal GABAergic projections to the LHb to provide feedback inhibition and encode reward. The DR provides serotonergic projections which inhibit excitatory innervation of the LHb from the EP, and itself generates excitatory postsynaptic currents.

As well as these firing modes, LHb function is characterised by its status as a highly interconnected relay structure mediating communication between the limbic forebrain and midbrain (Figure 3; Herkenham and Nauta, 1977, 1979; Sutherland, 1982). Most simplistically, the LHb recieves input from the hypothalamus and basal forebrain, integrates these signals, and exerts inhibitory control on monoaminergic centres in the midbrain. In reality, the nature of this connectivity is more complex, and is subject to extensive regulation. Potentially, the most influential factors driving daily variation in the LHb arises from its afferent connectivity with the SCN (Buijs, 1978; Zhang et al., 2009; Bano-Otalora and Piggins, 2017), the paraventricular nuclei (PVN; Hernandez et al., 2015), the dorsomedial hypothalamus (DMH; Ter Horst and Luiten, 1986; Morin, 2013), the lateral hypothalamic area (LH; Poller et al., 2013; Stamatkis et al., 2016) and the retina (Qu et al., 1996). Moreover, reciprocal connections with the Globus pallidus internus (GPi; Entopenduncular nucleus/EP in rodents; Shabel et al., 2012), ventral tegmental area (VTA; Omelchenko et al., 2009; Cerniauskas et al., 2019), the dorsal and medial raphe (DR, MR; Lima et al., 2017; Szonyi et al., 2019), and the rostromedial tegmental area (RMTg; Laurent et al., 2017; Tooley et al., 2018) are critical for the LHb to exert its role in the aetiology of depression. These connections will be explored more fully throughout this article (for a full review of LHb network connections, please see Hu et al., 2020).

## Intrinsic Regulation of Lateral Habenula Circadian Activity

Expression of the immediate early gene *c-fos* is conventionally used as a proxy for neuronal activity, and immunohistochemical detection of c-Fos protein facilitates assessment of such activity *ex vivo* (He et al., 2019). This tool is useful when determining if neuronal activity shows circadian variation and associating this with an animal's active behavioural state. In the rodent LHbM, c-Fos immunoreactivity (-ir) is positively correlated with activity levels, showing elevation during the behaviourally active night (Paul et al., 2011).

The potential explanations for this observation are myriad. It could mean that LHbM activity responds to behavioural feedback, that increased late day/nocturnal activity in the LHbM is regulated by the same networks which drive these temporal behavioural patterns, or that the LHbM possesses its own intrinsic rhythmicity. In support of the latter of these hypotheses, *ex vivo* electrophysiological studies revealed sustained day-night/circadian variation of neuronal activity in rodent LHb brain slices (Zhao and Rusak, 2005; Sakhi et al., 2014b). Since these LHb explants are isolated from extra-habenular circadian input, this suggests that these 24 h oscillations in LHb neurones are, at least in part, intrinsically generated.

In the SCN, and other circadian oscillators, intrinsic rhythmicity is generated by a “molecular clock.” This molecular clock is driven by a series of transcription/translation feedback loops (TTFL), featuring “clock genes” which encode transcription factors controlling the expression of highly diverse gene suites (for full review see Partch et al., 2014). The protein products of four integral clock genes drive the core TTFL: two activators (CLOCK and BMAL1) and two repressors (PER and CRY). Two homologues of *Per (Per1 and Per2)* and *Cry (Cry1 and Cry2)* exist and exhibit different distributions of expression across the mammalian brain (Shieh, 2003; Christiansen et al., 2016), and specifically within the habenula complex (Olejniczak et al., 2021).

Due to the rhythmic pattern of their expression, and their role in generating cell intrinsic rhythmicity, these clock genes can be used to investigate the independence of LHb oscillations. If, in *ex vivo* LHb explants, clock genes continue to show rhythmic expression then it suggests that oscillations in LHb activity undergo intrinsic regulation *via* the molecular clock. Assessments of clock gene expression, measuring bioluminescence from the PER2:Luciferase reporter construct, showed the maintenance of rhythmicity for up to 48–72 h in the *ex vivo* LHb brain slice (Guilding et al., 2010, 2013), while less robust, low amplitude rhythms are visualised in the adjacent MHb. Rhythms in PER2::LUC were most prominent in the LHbM, with a less distinctive signal detected in the LHbL. Similar to the SCN, the period of PER2::LUC rhythms in the LHb is elongated by the *Afterhours* mutation (Guilding et al., 2013), indicating that the molecular basis of circadian oscillations is conserved between the SCN and LHb. Indeed, *Per1*:Luciferase oscillations in the LHb explant are also observed (Sakhi et al., 2014b) and are lost in LHb slices from mice lacking a functional molecular clock.

There are some inconsistencies in the reporting of habenular clock gene expression, likely due to different approaches and experimental settings. When measured using *in-situ* hybridisation, *Per1* and *Per2* expression was observed to be more prominent in the MHb than the LHb (Olejniczak et al., 2021), while an earlier study suggested that clock gene expression was absent from the LHb (Shieh, 2003). Since Olejiniczak and colleagues combined regional markers in conjunction with real time PCR, when measuring *Per1* and *Per2* expression in the LHb, the most parsimonious conclusion is that expression of clock genes in the LHb is at a low level and may be localised to the LHbM which is adjacent to the lateral border of the MHb.

Daily changes in neurophysiological activity of LHb explants are also abrogated in *Cry1*^−/−^*Cry2*^−/−^ mice (Sakhi et al., 2014b). Since these mice do not have functional molecular clocks, the most likely explanation is that daily functional variation in LHb neuronal activity arises from intrinsic regulation by clock genes. However, it is also possible that other epithalamic/thalamic structures can entrain LHb activity and that it is the absence of rhythmic clock gene expression in these structures that underpins the loss in daily fluctuations in LHb neuronal activity. Interestingly, similar ablations of neural oscillations occur in the MHb of *Cry1*^−/−^*Cry2*^−/−^ mice (Sakhi et al., 2014a). It is therefore possible that functional circadian variations in the LHb are dependent on information flow from the MHb. Such an intrahabenular circadian circuit is evidenced by the existence of a unidirectional projection from the MHb to LHb (Kim and Chang, 2005), but the relevance of this requires further study.

## Extrinsic Regulation of Lateral Habenula Circadian Activity

As well as manifesting intrinsic regulation, *via* the molecular clock, the LHb sits within a diffuse neural circadian network featuring the SCN and various secondary oscillators (Bano-Otalora and Piggins, 2017). A large body of evidence suggests that the LHb receives either direct or indirect regulation from these timekeeping components. The presence of AVP-ir terminals in the LHb provides evidence for a putative projection from the SCN, as this neuropeptide is a highly expressed output molecule for the master clock (Buijs, 1978; Cagampang et al., 1994). Similarly, prokineticin 2 (PK2) projections from the SCN terminate in the LHb (Zhang et al., 2009); the LHb expresses the PK2 receptor (Zhou and Cheng, 2005), and PK2 evokes gabazine sensitive inhibitory currents to inhibit LHb neurones (Sakhi et al., 2014b). However, this PK2 projection is yet to be independently replicated, and AVP terminals in the LHb have since been traced to magnocellular neurones in the PVN (Hernandez et al., 2015). As such, the question of whether the SCN directly projects to LHb remains unresolved.

Nonetheless, the LHb likely receives indirect regulation from the SCN *via* other oscillators in the forebrain, thalamus and brainstem (Morin, 2013). For example, the SCN directly innervates the DMH and LH, which both show daily changes in neuronal activity (Marston et al., 2008; Guilding et al., 2009), and innervate the LHb (Poller et al., 2013; Stamatkis et al., 2016). This highlights polysynaptic pathways through which the SCN could indirectly regulate the LHb.

Alongside PK2 and AVP, LHb neurones are responsive to orexin (Flanigan et al., 2020; Wang et al., 2021). Orexin is a major output molecule of the LH (Aston-Jones et al., 2009; Richardson and Aston-Jones, 2012; Ferrari et al., 2018), and orexinergic neurones undergo circadian regulation from the SCN (Deboer et al., 2004; Marston et al., 2008; Kalsbeek et al., 2011). It is therefore possible that LHb dependent behaviours, driven by orexin, are under circadian control and that orexin is another mechanism by which daily variations in the LHb are extrinsically regulated. This is particularly relevant when considering the role of LH orexinergic neurones in driving appetitive drug seeking, a behaviour associated with reward dysfunction typical of aberrant LHb activity (James et al., 2019; Yeoh et al., 2019).

To complement this evidence for extrinsic circadian regulation of LHb activity, presynaptic release probability of LHb afferents varies throughout the day (Park et al., 2017). This peaks during late afternoon, coincident with increasing neuronal activity in the LHb (Zhao and Rusak, 2005; Sakhi et al., 2014b). This suggests that circadian variation in LHb activity is driven by an interaction between intrinsic and extrinsic regulation.

In addition to entrainment from the SCN and other secondary oscillators, evidence suggests that LHb activity could be influenced by light. An early tracing study evidenced a direct retinal projection to the LHb (Qu et al., 1996), suggesting that this structure could integrate photic information. Indeed, *in vivo* electrophysiological recordings show that LHb neurones respond to light (Zhao and Rusak, 2005; Sakhi et al., 2014b; Huang et al., 2019). However, later tract-tracing indicates that the retina innervates areas just external to the anatomical borders of the LHb (Sakhi et al., 2014b). Further, melanopsin expressing retinal ganglion cells (mRGCs), responsible for light entrainment of the SCN and other oscillators, do not directly target the LHb and instead innervate the adjacent perihabenula (PHb; Hattar et al., 2006; Morin and Studholme, 2014). Moreover, the delay in LHb response to retinal illumination is too slow to reflect a direct retina-LHb connection (Sakhi et al., 2014b), instead indicating that light information is conveyed to the LHb *via* polysynaptic connections.

Candidates for intermediate structures in this pathway include the ventral lateral geniculate and intergeniculate leaflet (vLGN-IGN; Huang et al., 2019). Viral tracing shows that these structures receive direct input from mRGCs, and optogenetic interrogation reveals that activation of this pathway drives inhibitory postsynaptic currents in LHb neurones. Moreover, *in vivo* electrophysiology reveals that retinal illumination decreases the action potential frequency, and burst frequency, of LHb neurones in an intensity dependent manner. Another investigation found varying levels of light intensities to increase mouse LHb neuronal activity (Sakhi et al., 2014b). These data highlight the uncertainty surrounding the predominant effect of light in regulating LHb activity. Light's role in entraining the LHb to daily rhythms has yet to receive extensive investigation.

Finally, it is worth considering a role for social cues in the regulation of this daily activity. The LHb is implicated in reward processing, and it is embedded within many pathways which regulate reward-based feeding (Stamatkis et al., 2016), drug seeking (Nair et al., 2021), and social behaviour (Valentinova et al., 2019; Rigney et al., 2020). It is possible that the LHb may receive feedback from the drivers of these behaviours—which accordingly entrain neural activity. Moreover, peak LHb neural activity *in vivo* is coincident with heightened physical activity levels during a rodent's subjective night (Paul et al., 2011), raising the possibility of a feedback loop entraining the LHb to physical exercise. Behavioural and SCN entrainment to such arousal and other non-photic cues is widely documented (Rosenwasser et al., 1984; Gillman et al., 2013; Crosby et al., 2019; Hughes et al., 2021; Robbers et al., 2021). As the LHb plays such a vital role in integrating this information and coordinating an animal's reward response (Lammel et al., 2012), it is possible that it also responds to variation in this information throughout the day. Interestingly, rhythmic expression of clock genes in the LHb can be dampened by voluntary consumption of a high-fat high-sugar diet, illustrating how food intake may regulate circadian rhythms in this structure (Blancas-Velazquez et al., 2017). However, contrasting research suggests that while this effect occurs throughout the brain, it is absent in the LHb (Blancas-Velazquez et al., 2018). Clearly, more research is required to resolve how hedonic eating, and other social cues, affect daily variation of molecular activity and function in the LHb.

## Implications for Psychiatric Illness

As previously discussed, the LHb is implicated in the pathophysiology of multiple psychiatric illnesses (Hu et al., 2020). Numerous depressive phenotypes, in various animal models, are associated with increased LHb activity. For example, elevated LHb spike frequency is correlated with depressive behaviour for mice in the tail suspension and sucrose preference test, following chronic mild stress (Cerniauskas et al., 2019). Conversely, increasing GABA_B1_ receptor function suppresses LHb hyperactivity and ameliorates depressive phenotypes (Lecca et al., 2016). This illustrates how bidirectional modulation of LHb activity can drive a two-way regulation of depressive behaviours.

This is likely due to the inverse correlation between LHb activity and the activity of its midbrain targets. The VTA receives direct innervation from the LHb and activation of this pathway promotes behavioural despair and place avoidance (Cerniauskas et al., 2019). Similarly, there is reciprocal connectivity between the LHb and the raphe nuclei (Sego et al., 2014); with stimulation of the LHb attenuating activity in the raphe (Stern et al., 1979). Paradoxically, though, almost all projections from the LHb are glutamatergic (Aizawa et al., 2012). Excitatory glutamatergic transmission to VTA and raphe neurones, predominantly dopaminergic and serotonergic, respectively, would be predicted to promote antidepressant-like effects. To reconcile this, the role of the RMTg must be considered (Barrot and Thome, 2011; Jhou, 2021).

The RMTg, also called the tail of the VTA, is a predominantly GABAergic midbrain centre which exerts feedforward inhibition onto various monoaminergic structures including the VTA, MR, and DR (Metzger et al., 2021). The LhB innervates the RMTg (Li et al., 2019), and c-Fos-ir in this structure is elevated following LHb stimulation (Lammel et al., 2012). Correspondingly, activation of the RMTg induces pro-depressive behavioural consequences (Stamatakis and Stuber, 2012; Smith et al., 2019), and reduces activity levels in the VTA and raphe nuclei.

The activity of the LHb, and thus feedforward inhibition of midbrain monoaminergic centres, is tightly regulated. Firstly, the VTA is reciprocally connected to the LHb, innervating the LHb with gabazine sensitive inhibitory synapses (Stamatakis et al., 2013). Activation of this pathway disinhibits the VTA *via* the suppression of the LHb, and thus the RMTg. This is one mechanism whereby a LHb-VTA circuit may mediate reward prediction error (Stopper et al., 2014). Elevated LHb activity is correlated with reward omission (Matsumoto and Hikosaka, 2007), and pathological LHb hyperactivity can therefore encode anhedonia typical of depression sufferers (Yang et al., 2018a).

The LHb also receives extensive innervation from the GPi (or rodent EP; Shabel et al., 2012). This glutamatergic projection promotes aversion learning, and optogenetic stimulation of the EP-LHb pathway conditions avoidance in the place preference test. Activity in this pathway is bidirectionally regulated by expected reward outcome, increasing and decreasing upon worse and better outcomes, respectively (Stephenson-Jones et al., 2016). Intriguingly, GABA and Glutamate can be co-released at the EP-LHb synapse, implicating release balance of these neurotransmitters in encoding mood and reward valence (Shabel et al., 2014).

Similar to the VTA, there is reciprocal connectivity between the LHb and the raphe nuclei (Metzger et al., 2021). Interestingly, despite the well-established role for serotonin signalling in the mechanism of traditional antidepressant drugs (Blier and Montigny, 1998), activation of 5-HT_2C_ receptors in the LHb causes depolarisation and increased spike frequency (Zuo et al., 2016). This is accompanied by the expression of an anhedonic and passive coping phenotype (Han et al., 2015). However, serotonin also decreases release probability of glutamate at the EP-LHb synapse, decreasing EPSP amplitude and potentially reducing excitatory input to the LHb (Shabel et al., 2012). By attenuating excitatory drive to the LHb, hyperactivity is suppressed, thus elucidating a potential mechanism whereby serotonin exerts its antidepressant effects.

There is accumulating evidence that increased LHb burst firing plays a role in the aetiology of depression (Cui et al., 2018; Yang et al., 2018a). For example, the proportion of bursting LHb neurones is elevated in congenitally learned helpless mice, compared to control animals. Indeed, optogenetic induction of LHb rebound bursting is sufficient to induce depressive-like behavioural performance in the sucrose preference and tail suspension tests (Yang et al., 2018a). This is demonstrative of a potentially causal relationship between this discharge pattern and depression.

This increase in LHb bursting is possibly associated with enhanced astrocytic expression of the inwardly rectifying potassium channel, Kir4.1 (Cui et al., 2018). Historically, glial expression of Kir4.1 has been colocalised with tripartite synapses (Newman, 1993), where its K^+^ buffering properties controls neuronal membrane potential according to the Nernst equation (Amédée et al., 1998; Neusch et al., 2006; Cui et al., 2018). Dysfunction of Kir4.1 results in local hyperactive neuronal states associated with various pathologies such as Parkinson's and epilepsy (Haj-Yasein et al., 2011; Tong et al., 2014; Nwaobi et al., 2016).

**Figure 4:**
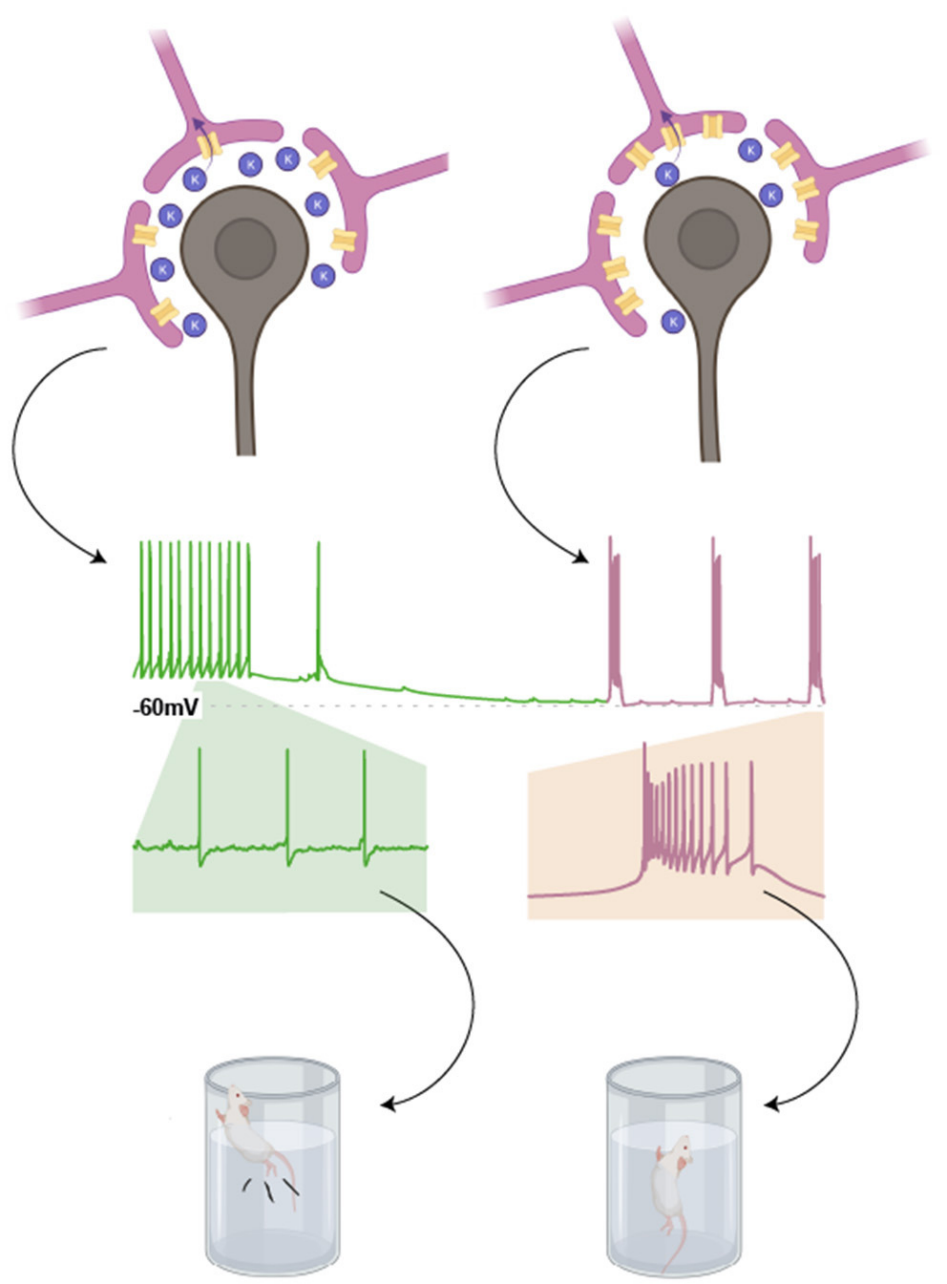
Astrocytic expression of Kir4.1 promotes burst firing in neighbouring LHb neurones. Expression of the inwardly rectifying potassium channel, Kir4.1, in astrocytic processes surrounding the somata of LHb neurones, lowers the concentration of K^+^ in the extracellular space. This is because K^+^ is taken up by these astrocytes. According to the Nernst equation, this results in a more negative resting membrane potential for neighbouring LHb neurones (as indicated in the cartoon trace). This in turn promotes burst firing in LHb neurones, a phenomenon associated with depressive phenotypes in rodent models (Drawn with BioRender).

In the LHb, these channels sit within the membrane of astrocytic processes that surround neuronal somata. In this context, Kir 4.1 drives the hyperpolarisation of neighbouring neurones by decreasing the extracellular concentration of K^+^ (Tong et al., 2014). LHb neurones require membrane hyperpolarisation to exhibit burst firing. As such, astrocytic overexpression of Kir4.1 promotes LHb burst firing, and consequent depressive phenotypes (Figure 4).

This link between depression and the voltage dependence of burst firing may explain the paradoxical nature of serotonin's depolarising effects in the lateral habenula (Zuo et al., 2016), and its well-characterised involvement in antidepressant pharmacotherapies (Blier and Montigny, 1998). By causing depolarisation in the LHb, serotonin may shift membrane potential out of a range in which burst firing occurs. In a similar fashion, knockdown of Kir4.1 causes depolarisation in LHb neurones (Cui et al., 2018). This is accompanied by attenuation of burst firing and alleviation of passive coping and anhedonic phenotypes in chronically learned helpless mice.

## Linking Circadian Variability With Depression, *via* the Lateral Habenula

Circadian disruptions are common in bipolar disorder and animal models of bipolar disorder (Wulff et al., 2010; Timothy et al., 2018) as well as in people suffering from depression (Jagannath et al., 2013; Mendoza, 2019). Many sufferers struggle to initiate or maintain sleep, and experience aberrant hormonal daily rhythms (e.g., cortisol; Difrancesco et al., 2019; Hoyos et al., 2020). Unpicking the cause-and-effect relationship of this comorbidity is complex, but the circadian and pro-depressive properties of the LHb make it a possible candidate to mediate this interaction.

Circadian variations in LHb activity are a good place to start when considering this interaction. LHb activity levels are closely linked to depression, therefore it would be predicted that peak exhibition of depressive symptoms to be coincident with elevated LHb firing rate. Mood does show diurnal variation, with negative affect showing more pronounced circadian rhythms in depression sufferers than healthy individuals (Wirz-Justice, 2008). Interestingly, the patterns of mood oscillations are different in healthy individuals compared to those exhibiting depressive mood (Rusting and Larsen, 1998). Low mood sufferers tend to experience an evening-worse pattern of mood, in which low mood is exacerbated in late day. However, some research indicates that, in diagnosed depressives, this peak in low mood occurs in early morning (Zerssen et al., 1985). This is coincident with the lowest behavioural activity in depressed adults over age 30 (Smagula et al., 2021). It is pertinent to note, though, that most recordings which illustrate circadian oscillations in the LHb were made in or from tissue prepared from nocturnal rodents. To fully appreciate how changes in LHb firing rate associate with daily variation in human mood, studies investigating neural activity variations in the LHb of diurnal rodents or indeed humans are necessary.

While circadian variation in spike rate has been independently investigated, it is unknown if the occurrence of burst firing or intraburst frequency alters with time of day/circadian cycles. This could have implications for symptoms of depression, as passive coping and anhedonia are shown to positively correlate with both of these variables (Yang et al., 2018a; Cerniauskas et al., 2019). Although circadian variation in the proportion of bursting LHb cells is not reported, Kir4.1 shows similar oscillatory expression to the clock gene *Bmal1* in retinal Müller cells (Luo et al., 2019). As astrocytic Kir4.1 expression is implicated in LHb bursting (Figure 3; Cui et al., 2018), it is possible that circadian oscillations in Kir4.1 drive a corresponding rhythm in burst firing and resultant depressive behaviours. Moreover, Kir4.1 expression is additionally regulated by insulin receptor substrate-1 (IRS-1; Luo et al., 2019), with increased IRS-1 signalling causing an upregulation of Kir4.1. Interestingly, the habenula complex is an enriched site for IRS-1 (Baskin et al., 1993). This suggests a potential mechanism whereby LHb activity is entrained by diet, with post-feeding surges in insulin causing increased IRS-1 signalling and thus changes in Kir4.1 expression (Crosby et al., 2019). Such a process could also explain how high-sugar diets may exacerbate depression (Vermeulen et al., 2017).

Circadian oscillations in LHb activity may entrain 24 h oscillations in the activity and function of neural structures that are targeted by LHb efferents (Mendoza, 2017). Some VTA neurones exhibit circadian variation in electrophysiological properties (Luo et al., 2008), and disruptions in these normal oscillations are associated with mood switching, typical of bipolar disorder (Sidor et al., 2016). The VTA shows circadian oscillations in clock gene expression (Webb et al., 2009), and is directly innervated by the SCN (Luo and Aston-Jones, 2009), both of which may entrain VTA function to its daily rhythms. However, LHb-VTA connectivity is another mechanism through which circadian variation in the VTA may occur. This particular pathway may be responsible for circadian oscillations in motivation and reward valency (Webb et al., 2009; Acosta et al., 2020). Aberrations in this process could explain the dysregulated reward seeking behaviour typical of addiction, or the anhedonia described in depression sufferers (Fox and Lobo, 2019). This is reinforced by the observation that arrhythmic animals show an overall decrease in motivation (Acosta et al., 2020).

Similar to the VTA, serotonergic raphe neurones appear to be under circadian control from the LHb (Mendoza, 2017). Serotonin release, throughout the brain, is heightened during the active night of nocturnal rodents (Dudley et al., 1998). There are some direct glutamatergic projections from the LHb to the DR, and electrical stimulation of these increase serotonin release (Kalen et al., 1989). Further, *in vivo* recordings of LHb neurones projecting to the DR show an elevated spike rate at night (Liu et al., 2021). This may explain the coincident peaks in LHb neural activity and serotonin release.

This circadian variation was blunted in stress susceptible mice, with an overall increase in activity at both day and night (Liu et al., 2021). Such stress susceptibility may be encoded by reciprocal serotonergic projections from the DR to the LHb, which thus increase LHb spike rate (Zuo et al., 2016). Alternatively, these recordings may have been taken from inhibitory pathways which inhibit serotonergic activity. To resolve these possibilities, recordings in postsynaptic raphe neurones are required.

Entrainment of LHb activity, by light, potentially underpins resistance to depressive mood. Bright light therapy (BLT) is an effective chronotherapy when administered in the early morning (Olejniczak et al., 2021), and is especially effective at treating seasonal affective disorder (Pjerk et al., 2020). In the forced swim test, passive coping behaviour is attenuated by transient light pulses during late night (Olejniczak et al., 2021). This suggests that light itself does not provide the antidepressant effect, but instead causes a circadian phase shift (Menculini et al., 2018). A putative pathway, connecting the retina to the vLGN-IGN and onward to the LHb, is thought to mediate the anti-depressive effect of BLT (Huang et al., 2019). Optogenetic activation of this pathway elicits an antidepressant effect in the sucrose preference and forced swim test, and these effects are replicated following retinal illumination. However, light suppresses active behaviour raising the possibility that observed changes are the consequence of a non-circadian “masking” effect (Redlin, 2001). Moreover, projections from the retina to the PHb are associated with mood regulation (Fernandez et al., 2018). Mice exposed to aberrant light conditions exhibit anhedonic and passive coping behaviours, and this is thought to be mediated *via* PHb neurones. This is another example of non-circadian response to light, near the habenula complex, which could explains light's regulation of affect.

An alternative explanation for the antidepressant-like effects of BLT is its capacity to induce *Per1* expression. This occurs in the SCN, and the LHb (Yan and Silver, 2002; Kuhlman et al., 2003; Olejniczak et al., 2021). More importantly, the capacity for BLT to elicit antidepressant-like effects appears to depend on *Per1* expression in the LHb and is abrogated by *Per1* knockdown in this structure. However, this may not be demonstrative of a causal relationship between *Per1* induction and antidepressant-like effects of BLT. *Per1* controls the transcription of various ion channels (Gumz et al., 2010; Stow et al., 2012; Alli et al., 2019) which regulate neuronal excitability (Carr et al., 2001; Lang et al., 2003; Amin et al., 2005). Upon *Per1* knockdown, these proteins become unregulated and the LHb could default to a state of hyperactivity. Interestingly, in the *Cry1*^−/−^*Cry2*^−/−^ mouse in which the molecular clock does not function, LHb spiking activity is around an intermediate level (Sakhi et al., 2014b), indicating that more investigation is necessary to determine if and how altered clock gene expression influences neuronal excitability.

Other studies support the contention that the molecular clock is involved in depression. For example, *Per2* knockdown in the LHb results in a passive coping phenotype during night-time (Li et al., 2020). Moreover, the antidepressant effects of ketamine have been associated with its impact on the molecular clock. Ketamine administration can dampen clock gene oscillations by interfering with the CLOCK:Bmal1 heterodimer (Bellet et al., 2011), which occurs in a GSK3β dependent manner. Ketamine's antidepressant effects are also dependent on GSK3β (Zanos and Gould, 2018), thus linking the molecular clock to depression. However, no behaviour experiments have yet shown a correlation between ketamine's effect on CLOCK:Bmal1 and depressive phenotypes.

If the exact nature of clock gene function in depression is questionable, their relevance in the entrainment of circadian oscillators is not. However, other cues are also important and may contribute to the link between circadian disruptions and depression. One example of this may be stress. Exposure to stressful stimuli can profoundly interrupt normal reward processing, a behavioural effect triggered by synaptic depression in the LHb (Nuno-Perez et al., 2021). Alongside this, acute stress is able to transform LHb reward responses into punishment-like signals, and this occurs synchronously with onset of anhedonic behaviour (Shabel et al., 2019). These stress signals can also cause circadian disruptions (Kalmbach et al., 2018). In fact, there has been an unprecedented rise in sleep disturbances as a consequence of the COVID-19 pandemic (Morin et al., 2020), an event which is concomitant with elevated depression and anxiety levels (Luo et al., 2020). The susceptibility of the LHb to stress, and its role as a neural circadian oscillator, places it well to mediate this link between stress dependent circadian disruptions and affective disorders.

In addition to stress, arousal-promoting stimuli such as physical exercise entrain circadian rhythms (Mistlberger and Antle, 2011; Hughes and Piggins, 2012). Scheduled exercise can entrain the SCN and behavioural rhythms in animals with otherwise disrupted activity patterns (Power et al., 2010; Hughes et al., 2021). There is also evidence of a feedback loop coordinating LHb activity with exercise (Paul et al., 2011), and animals with LHb Per1 knockdown exhibit blunted behavioural rhythms (Li et al., 2020). Lower levels of exercise are associated with depression (Mcmahon et al., 2017), and exercise-based interventions can alleviate symptoms in sufferers (Cramer et al., 2013; Paolucci et al., 2018; Schuch and Stubbs, 2019). It is possible that there is a circadian association between depression and exercise, and that timed exercise intervention may benefit depression sufferers by entraining their otherwise disrupted diurnal rhythms (Hughes et al., 2021). If this this proves to be the case, the LHb may be a critical component in the neural circuitry which underpins it.

## Conclusion

The LHb varies its activity over 24 h, and this epithalamic area is ideally situated to mediate the links between depression and circadian disruption. It is a highly heterogeneous structure that resides within an anatomically diffuse neural circadian network, potentially receiving circadian entrainment signals from other neural oscillators, arousal, and social cues, as well as light input. The LHb's role in the regulation of reward processing, and the function this has in entraining rhythms according to motivation, evidences a connections between these rhythms and mood. Aberrant and dysregulated reward seeking is characteristic of various psychiatric disorders, and disruptions in LHb signalling may underpin this.

Extensive further research is necessary before firm conclusions can be drawn regarding the importance of changes in the circadian function of the LHb in the aetiology of depression. To date, most of the evidence supporting this conjecture is of a correlational or associative nature and here we have highlighted potential avenues for future investigation. Indeed, preliminary work is emerging which focusses on the role of circadian oscillations in LHb-dependent depression and antidepressant treatments. While important to understanding depression, such approaches need to be carefully critiqued to fully elucidate the complexity of the LHb's role in both depression and circadian timekeeping in general.

## Data Availability Statement

The original contributions presented in the study are included in the article/supplementary material, further inquiries can be directed to the corresponding author.

## Author Contributions

CY and HP wrote and edited the manuscript. DL edited the manuscript. CY and DL composed the figures. All authors contributed to the article and approved the submitted version.

## Funding

CY was supported by a studentship from the Biotechnology and Biological Sciences Research Council (BBSRC; UK) South West Biosciences Doctoral Training Programme (BB/T008741/1). Research in HP's lab was supported by project grants from the BBSRC (BB/R019223 and BB/W000865).

## Conflict of Interest

The authors declare that the research was conducted in the absence of any commercial or financial relationships that could be construed as a potential conflict of interest.

## Publisher's Note

All claims expressed in this article are solely those of the authors and do not necessarily represent those of their affiliated organizations, or those of the publisher, the editors and the reviewers. Any product that may be evaluated in this article, or claim that may be made by its manufacturer, is not guaranteed or endorsed by the publisher.
